# Characterisation of Goal Scoring Patterns during Open Play Related to Zone Pitch Division and Number of Players Involved in the 2018 FIFA World Cup

**DOI:** 10.3390/s21165601

**Published:** 2021-08-19

**Authors:** Joaquin Cerda, Javier Sanchez-Sanchez, David Viejo-Romero, Luis Jimenez-Linares, Jesus Vicente Gimenez, Jorge Garcia-Unanue, Jose Luis Felipe

**Affiliations:** 1 Faculty of Sport Sciences, Universidad Europea de Madrid, 28670 Madrid, Spain; joaquin.cerda@universidadeuropea.es (J.C.); david.viejo@universidadeuropea.es (D.V.-R.); joseluis.felipe@universidadeuropea.es (J.L.F.); 2 Department of Information Technologies and Systems, School of Computer Science, University of Castilla-La Mancha, 45071 Toledo, Spain; luis.jimenez@uclm.es; 3 Universidad Internacional de Valencia (VIU), 46002 Valencia, Spain; intertato_@hotmail.com; 4 Faculty of Health Sciences, Universidad Isabel I, 09003 Burgos, Spain; 5 IGOID Research Group, Department of Physical Activity and Sport Sciences, University of Castilla-La Mancha, 45071 Toledo, Spain; Jorge.GarciaUnanue@uclm.es

**Keywords:** types of goals, pitch division, involved players, machine learning, football

## Abstract

The aim of this study was to characterise all the goal scoring patterns during open play (elaborate attacks versus counterattacks) related to zone pitch division and the number of players involved in the 2018 FIFA World Cup in Russia. An Iterative Dichotomiser 3 (ID3) decision tree algorithm was used to classify all the goal scoring patterns (94 goals in 64 matches). The results did not show statistical differences between the type of scoring goal during the 2018 FIFA World Cup (*p* > 0.05; ES = Moderate). According to the result of the patterns of how the goals were achieved, an ID3 algorithm decision tree with seven classification decision nodes was calculated. Consequently, this study may aid national team coaches for the next World Cup to establish notational analyses and spatial-temporal relations to understand how scoring patterns during open play are related to zone pitch division and the number of players involved.

## 1. Introduction

Every four years, the International Federation of Association Football (FIFA) holds the FIFA World Cup. The event is the sport’s premier tournament, attracting the most elite teams from around the world [1]. To put this into perspective, the 2018 FIFA World Cup was watched by 3.5 billion people across the globe [2]; therefore, the FIFA World Cup is one of the biggest sports events. Given the rapid increase in technological and scientific studies as well as the sport reaching a wide range of locations, a large number of people have an interest in this area [3]. In recent years, football match analyses have helped increase knowledge about the sport’s physical, physiological and technical demands [4], providing key information about players’ performance during games [5] or different tournaments and competitions [6].

In this sense, match analyses in team sports and performance prediction can provide an objective, unbiased and valid record of team activities, and thereby help monitor and evaluate team performance [7]. Coaches and performance analysts look for the critical performance feature, to diagnose past performance (what happened and why) and attempt to predict and prescribe future behaviours (what will happen and what should be done) [8].

It is necessary to analyse elite football beyond the specific context of professional clubs, where training programmes, logistical demands and available facilities differ greatly from those in competitions involving national teams, such as the FIFA World Cup [9]. Nevertheless, previous literature has focused on analysing the risk of injury generated in the FIFA World Cup with respect to club competitions [10]. Moreover, most of the studies have analysed club competitions (national tournaments, the UEFA Champions League, etc.), whereas analyses of national team competitions remain limited [11,12].

Furthermore, although the match outcome is a primary criterion for evaluating performance in team sports, the closeness of the game (winning and losing margin) provides additional contextual information about the tactical and technical success of the competing teams [7,13,14]. Therefore, analysing the specific characteristics of the score directly determines the factors that ultimately lead to successful attempts and objectives in elite football [15].

Although plenty of research has examined whether goal scoring is affected by time in football clubs [15,16], other studies have focused on predictive modelling, employing data normalisation and regression analyses to explore the probability of goal scoring [17]. However, few studies have analysed the influence of goal scoring patterns on the results of national team competitions [18], and to date, none have used machine learning systems to explain the goals scored during national team tournaments.

Therefore, using machine learning, the current study aimed to characterise all the goal scoring patterns during open play related to zone pitch division and the number of players involved in the 2018 FIFA World Cup. It was hypothesised that during the 2018 FIFA World Cup, the teams will have scored more goals by elaborate attacks rather than counterattacks.

## 2. Materials and Methods

### 2.1. Match Sample and Data Source

Match samples and data were collected regarding 94 of the goals scored (nine goals were excluded because pitch-coordinates could not be determined due to players being in close proximity to each other) across the 64 games at the group and knockout stages of the FIFA World Cup held in Russia from 14 June to 15 July 2018. Data were retrieved from the private company Mediaset through its streaming platform. Videos of all the goals scored during the tournament were analysed using Kinovea (v.0.8.15; Kinovea Org., San Francisco, CA, USA) software through the ‘perspective grid’ tool. The programme used was identified to have reached an acceptable level, being suitably precise and reliable (both inter- and intra-rater) for use in the scientific field and providing an acceptable level of accuracy in angular and linear measurements obtained via the digitisation of *x*- and *y*-axis coordinates [19].

Ethical clearance was approved by the Ethical Committee of the European University of Madrid (CIPI04/2019).

### 2.2. Performance Variables

**Figure 1:**
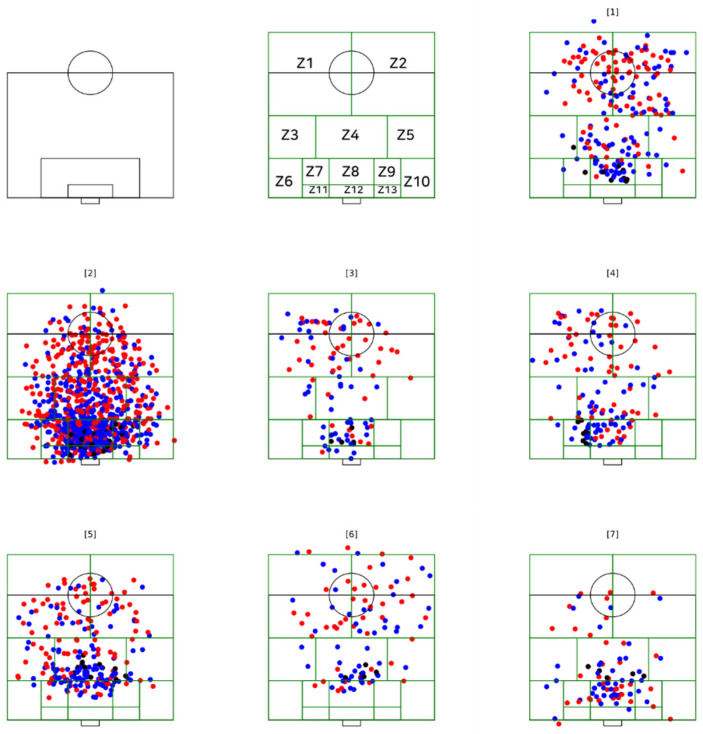
Plot space of use (pitch zone, 1 to 13) and distribution of players (attackers and defenders) was created in a numerical computing environment, by node (**1** to **7**), involving attackers and defenders in each goal scoring-related zone pitch division. Note on colours: red (attackers), blue (defenders) and black (goal scorers).

The analysis of variables coded in the study consisted in: (i) the frequency of different types of goals scored from open play [20]; (ii) zone pitch division (13 zones); and (iii) number of players involved, measured by the number of players occupied in relation to each goal scored (Figure 1).

Operational definitions of match variables were defined according to the available literature:

- **Team possession type:** Degree of offensive directness by the level of utilisation or the creation of imbalances in the opponent’s defence to achieve penetration (how quickly penetration is attempted after winning the ball). Penetration is achieved when a pass goes towards the opponent’s goal, past the opponent player(s), while the attacker(s) maintain a high degree of control over the ball. (i) A counterattack (‘direct play’) starts with winning the ball in play and progresses by either (a) utilising or attempting to utilise a degree of imbalance from start to end or (b) creating or attempting to create a degree of imbalance from start to end by using an early (first or second, evaluated qualitatively) penetrative pass or dribble. Utilising a degree of imbalance means seeking penetration in such a way that the defending team will fail to regain a high degree of balance from the start to the end of the attacking team’s possession. Counterattacks progress relatively quickly. (ii) An elaborate attack (‘possession play’) starts with winning the ball in play and progresses either (a) without utilising or attempting to utilise a degree of imbalance or (b) by creating or attempting to create a degree of imbalance by using a late (third or later, evaluated qualitatively) penetrative pass or dribble. Not utilising a degree of imbalance means seeking penetration in such a way that a defending team manages to regain a high degree of balance before the end of the attacking team’s possession. Elaborate attacks often progress relatively slowly [20].
- **Space of use (pitch zone):** The pitch was divided into 13 zones (from Z1 to Z13), splitting the pitch into four transversal zones parallel to the halfway and goal lines using six horizontal lines (see Figure 1). The pitch zones were adapted from the traditional nine-zone pitch division in accordance with Grehaigne et al. [21] to ensure sufficient accuracy of the zone code, where any spatial pitch zone is related to goal scoring (the last action performed by the player scoring the goal at the moment of shooting).
- **Number of players involved, measured by the number of players occupied in relation to the pitch zone from where a goal was scored:** The number of players participating (attackers and defenders) on each team at the time when each goal was scored.

### 2.3. Reliability Testing

An intra-observer test using the kappa measure of agreement was undertaken to assess the reliability of two types of goal scoring patterns. From the raw data in the spreadsheet, the test calculated an intra-rater agreement statistic (Kappa) that was evaluated for 94 goals scored from open play, of which 15 (15.9%) were randomly selected, analysed and re-analysed by the investigator after three weeks in order to reduce the learning effects [22]. The kappa values ranged from 0.86 to 1.00 (0.95), demonstrating that the strength of agreement was very good [23] for reinforcing the identification of different types of goal scoring patterns.

### 2.4. Procedure and Statistical Analysis

For the types of goal scoring patterns during open play related to zone pitch division in the dataset, normality was confirmed via the Shapiro–Wilk test. Comparisons between different types of goals and seven nodes were performed using Student’s *t*-test, with *p* < 0.05 considered a significant effect. Effect size (ES) values were estimated using Cohen’s d with the following criteria: >0.2 (small); >0.6 (moderate); and >1.2 (large) [24]. All data were analysed using the Statistical Package for Social Sciences (SPSS) v 25 (IBM Coorp, Chicago, IL, USA).

**Figure 2:**
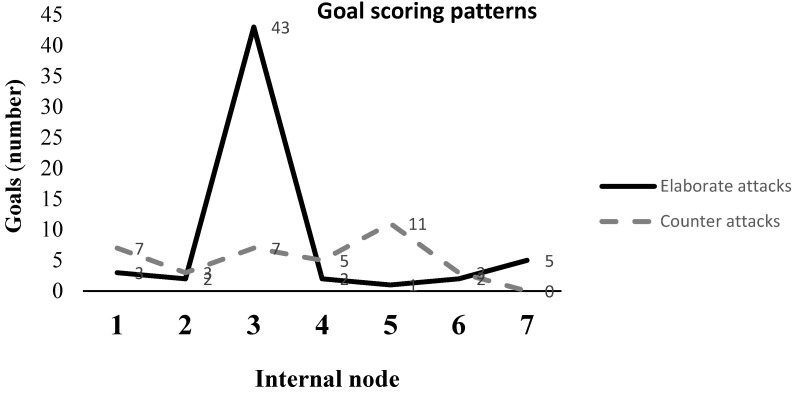
Number of goals and internal node.

**Figure 3:**
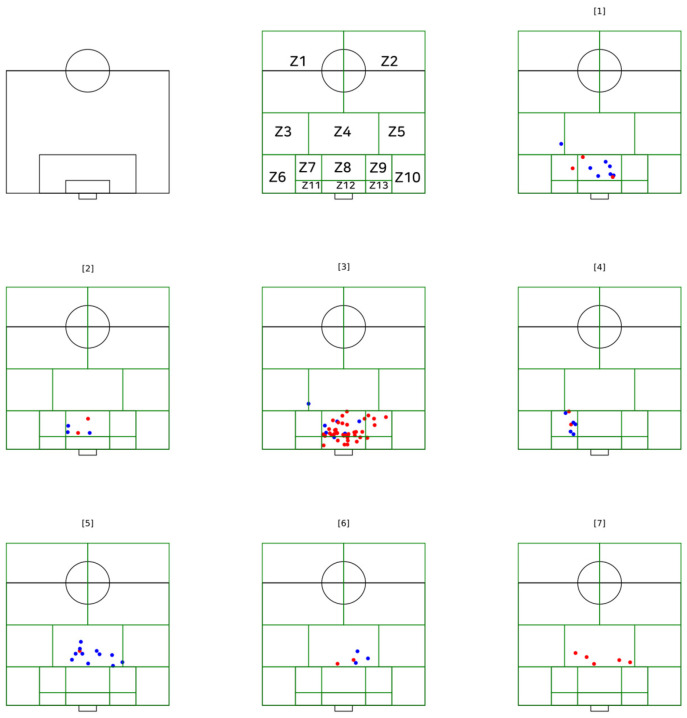
Plot of the number of goals scored in seven nodes. Note on colours: red (elaborate attacks) and blue (counterattacks).

**Figure 4:**
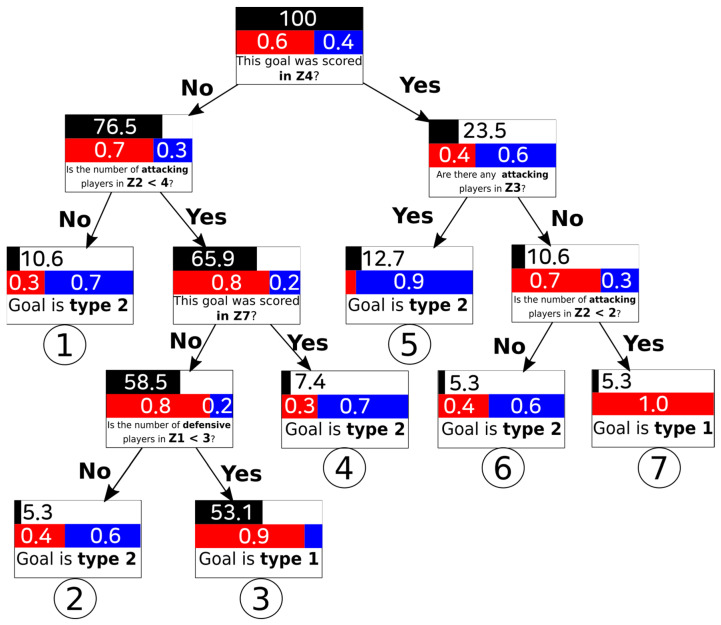
Final decision tree (Accuracy 0.83). Note on colours: black (% goal-scoring); probability of scored of different type goals: red (elaborate attacks) and blue (counterattacks).

In Figure 2, the goal scoring patterns are shown. This figure shows the seven nodes and the total number of goals scored (i.e., elaborate attacks and counter attacks) inside each node (1 to 7, extracted from the decision tree, Figure 3 and Figure 4).

The description of the goal is based in the outcome function plot (x, y) of the characterisation of the types of goal scoring patterns related to the zone pitch division and goal type using open-source programming language Python v 2.5 (Beaverton, OR, USA) using libraries for computer vision and machine learning, python-matplotlib, based on previous observations (Figure 3).

To provide the scientific community with a simple, accurate answer to the main research question, the algorithm Iterative Dichotomiser 3 (ID3) for the construction of a decision tree classification was used to extract rules and create a model able to predict the values of the target variables—goal scoring patterns (elaborate attacks and counterattacks), zone pitch division and the number of players involved—to handle the cut-off point values between categorical and numerical data. For the inference of the decision tree, 30% of the data has been used for testing and the remaining 70% as learning data. The set of tests and learning have been obtained randomly, maintaining the distributions of the two types of goals in both sets. The dataset was analysed using Rapidminer Studio (v 8.1, RapidMiner, Inc. Headquarters, Boston, MA, USA).

## 3. Results

The descriptive statistics (mean ± SD) showed 8.3 ± 5.9 goals were scored in elaborate attacks and 5.1 ± 3.5 were scored during counterattacks. These results did not show statistical differences between the type of scoring goal during the 2018 FIFA World Cup (*p* > 0.05; ES = Moderate).

According to the result of the patterns of how the goals were achieved (elaborate attack and counterattack), the number of players (attackers and defenders) and the occupied areas (zone 1 to zone 13), an ID3 algorithm decision tree with seven classification decision nodes was calculated (Figure 4).

## 4. Discussion

This machine learning-based study was conducted to characterise all of the patterns of the goals scored in open play during the 2018 FIFA World Cup in Russia related to the zone pitch division and the number of players involved. The findings showed that 60% of the goals were the result of elaborate attacks. This percentage is congruent with recent research by Kubayi [25] and with the findings of Wright et al. [26] and Njororai [27], showing that most goals come from short possession. This scoring rate is in line with the results showing that most goals came from short possession [26] due to short passes, offering a reliable way of advancing the ball to the opponent’s goal [28]. For all these reasons, the goal scoring pattern is a critical variable for a team’s success [29]. Furthermore, the decision tree (ID3) algorithm showed through leaf node3 that the most goals (43) were scored through elaborate attacks.

The fact that most of the goals of the 2018 FIFA World Cup were achieved through elaborate attacks could be attributed to the short time selections for the preparation of this event. It may be easier to train scoring situations through elaborate attacks. Thereby, coaches can devise an overall plan to attack in an elaborate way, balancing the space to avoid being surprised by counterattacks. Previous studies have shown that the most successful method of scoring goals is to pass the ball behind the opposing back line so that an attacking player can advance beyond the last defender and either shoot or pass the ball to a teammate to score in the penalty area [30], in line with the present research’s results.

In addition, our results illustrated that the goals during the tournament were related to zone pitch division and the number of players involved. It was evidenced that most of the goals that were scored (53%, with 0.9 through elaborate attack) had the same registered pattern, drawn in Node 3. Moreover, it is important that the number of attackers were <4 and the number of defenders were <3. In this sense, the goals scored with this pattern could be associated to the fact that there is a lot of free space to use [31]. Consequently, football coaches may pay more attention to practising the rational use of space and the number of players involved, employing provocation rules in order to encourage the use of short passes to advance the ball in a way that causes the opponent to lose control of the ball [28]. Our results are also in line with the findings by Gómez-Ruano et al. [32], as they revealed a higher probability of scoring a goal, having recovered the ball in the middle of the field and organising attacks towards the penalty area to increase the number of shots, and consequently, goals.

In nodes 1, 4 and 5, the pattern of successful score goal is the counterattack. This is determined by the number of attacking players, demonstrating that most goals were scored from open play. Consequently, national team coaches should protect Zone 2 (according to node 1), Zone 7 (node 4) and Zone 3 (node 5).

Therefore, these findings suggest that the most goals are generated in the last third and came from elaborate attacks. These results provide a new approach to goal scoring in football and exemplify a novel method to determining the prominent number of players and pitch zones involved through the different nodes obtained by the ID3 algorithm. Methods such as social network analysis and notational analysis have been implemented to examine goals and passing networks in previous studies [33], but this has been the first investigation to focus on machine learning to extract rules and create a model that can predict the values of the target variables.

While the results of this study provide new information regarding the scored the type of goals, space of use (pitch zone) and number de player involved during World Cup 2018, these findings must be considered in light of a number of limitations. Firstly, the pass number (short, medium and long possession) was not considered. Secondly, the pass length (short, long and mixed passes) was not examined. Thirdly, these variables were not analysed in combination with tactical formation during the matches, which could modify the way of scoring goals patterns during the 2018 FIFA World Cup and future tournaments. Thus, future research should analyse these aspects. Finally, the current information is related to the 2018 World Cup, and these findings may not be applicable to other populations.

## 5. Practical Implications

This type of analysis is important for identifying the goal scoring patterns during open play related to the zone pitch division and number of players involved in the 2018 FIFA World Cup. An analysis of this kind provides useful information to demonstrates that goals were scored from elaborate attacks, in the final third and that the number of attackers was <4 and the number of defenders was <3. For this, both soccer coaches and sports scientists should pay more attention to practicing the following during their training session: integrating tasks with objectives of short possessions, playing fast and shooting on target in order to yield more goal-scoring opportunities.

## 6. Conclusions

A decision tree is a useful tool for precisely laying out the relative numbers of different types of goals, based on the zone pitch division of the shooter and the number of players involved in an attack. This may suggest that the goal scoring efficiency of the teams competing in the 2018 FIFA World Cup was dependent on possession type. In summary, this study’s main finding is that most of the goals scored during this event resulted from elaborate attacks, the final third related to pitch zones 8 and 12 and during the situations with <4 attackers and with <3 defenders (node 3).

The nodes identified here are of great relevance to soccer scientists and coaches, as they can identify goal scoring patterns. For this reason, the ID3 algorithm can be regarded as a reliable method for identifying the zones and the number of players involved in the majority of goals. To this end, there is a need for coaches to change their perspectives and to include analyses and spatial-temporal relations in order to understand scoring patterns during open play related to zone pitch division and the number of players involved.

## Data Availability

All data were extracted from the videos of the matches broadcast by Spanish TV during 2018 FIFA World Cup.
